# Visual Sensory Experiences From the Viewpoint of Autistic Adults

**DOI:** 10.3389/fpsyg.2021.633037

**Published:** 2021-06-08

**Authors:** Ketan R. Parmar, Catherine S. Porter, Christine M. Dickinson, James Pelham, Peter Baimbridge, Emma Gowen

**Affiliations:** ^1^Division of Neuroscience and Experimental Psychology, School of Biological Sciences, Faculty of Biology, Medicine and Health, Manchester Academic Health Science Centre, The University of Manchester, Manchester, United Kingdom; ^2^Division of Pharmacy and Optometry, School of Health Sciences, Faculty of Biology, Medicine and Health, Manchester Academic Health Science Centre, The University of Manchester, Manchester, United Kingdom; ^3^Greater Manchester Autism Consortium, Manchester, United Kingdom; ^4^Autscape, Coventry, United Kingdom; ^5^Autism@Manchester, Manchester, United Kingdom; ^6^Salfordautism, Manchester, United Kingdom

**Keywords:** autism spectrum conditions, vision, visual sensory experiences, altered sensory reactivity, focus groups, qualitative methods, autistic adults, coping strategies

## Abstract

Although previous research has investigated altered sensory reactivity in autistic individuals, there has been no specific focus on visual sensory experiences, particularly in adults. Using qualitative methods, this study aimed to characterize autistic visual sensory symptoms, contextualize their impact and document any associated coping strategies. A total of 18 autistic adults took part in four focus groups which involved questions around visual experiences, the impact of these on daily life, and strategies for their reduction. Transcripts of each session were thematically analyzed allocating six key themes. Participants described a range of visual hypersensitivities, including to light, motion, patterns and particular colors, which contributed to distraction and were frequently part of a wider multisensory issue. Such experiences had significant negative impacts on personal wellbeing and daily life with participants describing fatigue, stress and hindrances on day-to-day activities (e.g., travel and social activities). However, the degree of understanding that participants had about their visual experiences influenced their emotional response, with greater understanding reducing concern. Participants employed a variety of coping strategies to overcome visual sensory experiences but with varied success. Discussions also highlighted that there may be a poor public understanding of sensory issues in autism affecting how well autistic individuals are able manage their sensory symptoms. In summary, autistic adults expressed significant concern about their visual experiences and there is a need to improve understanding of visual experiences on a personal and public level as well as for developing potential support.

## Introduction

In addition to social interaction and communication difficulties, altered sensory reactivity, such as excessive (hyper-) or dampened (hypo-) sensitivity to stimuli, forms part of the autism diagnostic criteria (DSM-5: American Psychiatric Association, 2013; ICD-11: World Health Organization., 2019). Hypersensitivity describes an increased response such as extreme light or sound sensitivity whereas hyposensitivity describes an obviously dampened response including apparent increased pain and temperature thresholds. In addition, individuals can also exhibit unusual interests in sensory aspects of the environment, such as excessive touching of object edges or fascination with reflections (Simmons et al., 2009; DSM-5: American Psychiatric Association, 2013).

Altered sensory reactivity is experienced by the majority of autistic people (Kientz and Dunn, 1997; Green et al., 2016). These experiences can be enjoyable or distressing (Smith and Sharp, 2013; Robertson and Simmons, 2015), and their magnitude has been found to be positively correlated with the number of autistic traits one may have (Robertson and Simmons, 2013). There is debate around whether altered sensory reactivity increases (Liss et al., 2006) or decreases (Kern et al., 2006) with age. Importantly, it remains throughout life (Crane et al., 2009) and affects each modality (Cléry et al., 2013; Baum et al., 2015) as well as multisensory processing (Marco et al., 2011; Beker et al., 2018).

The current study focused on visual sensory experiences of autistic adults. Informal discussions between autistic individuals and members of the research team, prior to this study, revealed the multidimensional difficulties that visual sensory experiences could cause for many autistic people, leading to them trying to manage these sensory issues themselves. This was exemplified by many autistic community members accessing unregulated ‘‘treatment’’ options such as tinted lenses which are claimed to be suitable management options^1^ although there is no evidence base for this. Additionally, research has shown autistic individuals to present frequent ophthalmic conditions such as altered binocular vision, strabismus, refractive errors and compromised retinal structure (Little, 2018). It is possible that these conditions may be linked to autistic visual sensory experiences, but further research is needed. To be able to investigate this possible link, there is first a need to fully characterize visual sensory experiences together with their impacts on autistic people.

Bogdashina (2003) provided a list of visual hypersensitivity issues, such as focusing on fine detail and a dislike for extreme or flashing lights, and hyposensitivity issues, such as fascination of reflections or colorful objects and intensely focusing on objects or people. However, few studies have provided further characterization of visual sensory issues in autistic individuals, particularly in adults. Autistic visual hypersensitivities have been found to overlap with key characteristics of Meares-Irlen syndrome, also known as visual stress (Wilkins, 1995, 2003), defined as visual discomfort as a result of an increased sensitivity to repetitive patterns.

Subjective altered sensory reactivity in autism has been mostly explored using questionnaires. Findings from a recent meta-analysis of 55 questionnaire studies across children and adult populations (Ben-Sasson et al., 2019) supported the atypical nature of sensory symptoms in autistic individuals and highlighted the most consistent sensory experience was hypersensitivity. Whilst confirming heightened sensitivity across all sensory modalities (Tavassoli et al., 2014a,b), questionnaire studies have also made clear that the severity of sensory sensitivity varies between individuals (Ben-Sasson et al., 2008; Crane et al., 2009; Elwin et al., 2017). Of these studies, it is only Tavassoli et al. (2014a, b) who highlight the importance of investigating individual modalities so to not obscure intramodality differences. Specific to vision, they reported autistic adults to display heightened sensory sensitivity (Tavassoli et al., 2014a) and greater hypersensitivity (Tavassoli et al., 2014b) to visual stimuli relative to controls.

Quantitative research can be complemented and expanded upon by qualitative research. Although questionnaire methodology has provided extensive data about altered sensory reactivity in autistic individuals, it restricts the extent to which participants can express themselves and limits understanding of how experiences for each sensory modality may differ. Comparatively, qualitative techniques (e.g., focus groups or interviews) allow researchers to explore new ideas to greater depth and in different dimensions, such as attitudes, social interaction, thoughts and meaning (Malterud, 2001).

Qualitative studies have provided detailed evidence for general altered sensory reactivity in autistic individuals, although none have focused on visual sensory issues. Kirby et al. (2015) used semi-structured interviews to investigate sensory experiences in autistic children. Experiences were generally described as “likes or dislikes” with interviewers unable to determine sensory issues within individual modalities. They concluded this to indicate that autistic children view their experiences as multisensory. Altered sensory sensitivity amongst autistic adults has been documented using semi-structured interviews (Smith and Sharp, 2013), focus groups (Robertson and Simmons, 2015), and analysis of personal accounts (Jones et al., 2003), but this was not explored for individual modalities. However, some visual experiences were superficially reported including difficulties tolerating a range of stimuli such as bright environments, artificial lighting, patterns, unpredictable movements, visual distractions, fine detail, and particular colors (Jones et al., 2003; Smith and Sharp, 2013; Robertson and Simmons, 2015). Child group interviews by Robertson (2012) revealed similar visual difficulties; some colors, bright lights and screens, and additionally certain shapes can cause painful sensations.

The impact of general altered sensory reactivity on autistic individuals has previously been investigated revealing negative and pleasurable emotions, negative physical symptoms, effects on attention, and both positive and negative impacts on daily living (Jones et al., 2003; Smith and Sharp, 2013; Robertson and Simmons, 2015). Strategies to cope with these include purposeful exposure to positive stimuli or avoiding, accommodating, distracting away from, and seeking the positive aspects in negative stimuli (Jones et al., 2003; Smith and Sharp, 2013; Robertson and Simmons, 2015). In an interview study about autistic adults’ daily lives, Robledo et al. (2012) found participants to mention that although visual stimuli could cause negative emotions and physiological responses, certain lighting or color combinations could be enjoyable.

While these qualitative studies have provided a superficial description of visual experiences, there are no studies that have specifically examined autistic visual sensory experiences in depth. General findings regarding impacts of sensory experiences on quality of life and coping strategies cannot be assumed to apply across every sense. Studies which have documented subjective visual experiences (Jones et al., 2003; Robertson, 2012; Robledo et al., 2012; Smith and Sharp, 2013; Robertson and Simmons, 2015) have not attempted to explore these further or characterize them in-depth but instead summarize them broadly alongside other modalities.

On the other hand, a large body of work has examined vision using cognitive and psychophysical tasks in autistic people (Simmons et al., 2009; Schauder and Bennetto, 2016; Apicella et al., 2020; Federici et al., 2020). Various studies have investigated performance of autistic and non-autistic individuals in tasks linked to early visual processing, such as visual acuity (Tavassoli et al., 2011; Albrecht et al., 2014; Tebartz van Elst et al., 2015) and contrast sensitivity (Koh et al., 2010). Higher level visual processing has also been explored, for example, face recognition (Tang et al., 2015) and global (Van der Hallen et al., 2015) and biological motion perception (Todorova et al., 2019). While findings are mixed, these studies suggest fewer group differences for lower than higher level visual processing. Autistic people have also been found to exhibit perceptual differences, with a superiority or preference in processing local compared to global information (Plaisted et al., 1999; Rinehart et al., 2000; Happé and Frith, 2006; Mottron et al., 2006; Simmons et al., 2009; Muth et al., 2014; Kabatas et al., 2015).

It is evident that whilst qualitative studies have provided detailed evidence for general altered sensory sensitivity in autistic individuals, the visual sense has not been explored to the same extent with no previous studies focusing specifically on subjective visual sensory experiences. Moreover, these studies have been conducted mainly from a psychology or psychophysics point of view; how would a vision and ocular health expert interpret these findings? This is an important gap to fill as improved descriptions of visual sensory issues, as well as from a different professional perspective, can suggest directions for future quantitative studies. For example, if descriptions of autistic visual sensory issues overlap with characteristics of visual stress, binocular vision symptoms, poorly corrected refractive error or are more suggestive of cognitive mechanisms (Happé and Frith, 2006; Mottron et al., 2006; Van de Cruys et al., 2014), future work can be targeted to test these links. Additionally, it is clear from previous reviews (Schauder and Bennetto, 2016; Ben-Sasson et al., 2019) that there has been a greater focus on investigating sensory difficulties in autistic children than adults. In Ben-Sasson et al.’s (2019) meta-analysis, only 7% of questionnaire studies that examined sensory symptoms involved adults. Overall, a detailed characterization of the multi-faceted visual sensory experiences in autistic adults, the specific impacts of these on daily life and the strategies employed to cope with these does not exist. The aim of the current study was to gain a detailed insight into the everyday visual experiences of autistic adults along with their impact and coping strategies employed, from the point of view of an optometrist.

A qualitative approach was taken in order to explore the full range of visual experiences that autistic people report and to what extent these impact their daily lives. Focus groups were employed as they allow opinions to be collated from a relatively larger sample, compared to one-to-one interviews, and have been successfully conducted with autistic adults in previous research (Robertson and Simmons, 2015; John et al., 2017; Koffer Miller et al., 2017; Gowen et al., 2019). Furthermore, interactions between members in a focus group allow researchers to understand the range of opinions as well as the level of agreement about topics (Barbour, 2008), particularly suitable for the current study’s aims.

## Materials and Methods

### Recruitment and Participants

An advert was publicized by email and social media using the Autism@Manchester network, local autism groups and the university platforms. Flyers were also displayed around the university campus and handed out at autism events. Inclusion criteria were (i) being formally diagnosed as autistic; (ii) absence of a learning disability; (iii) aged 18 years or above; (iv) being able to travel to the university, and; (v) availability to attend one of the specified focus group sessions.

An opportunity sample was recruited for this study. Although 27 participants signed up to a focus group session, nine did not attend. A total of 18 autistic adults took part, aged 25 to 67 years (mean age 47.1 years), of which six were female. All had a formal diagnosis of an autism spectrum condition (autism/Asperger’s syndrome/ASC) visually confirmed by a diagnosis letter, and were from the northern regions of England. In terms of ocular history:

• 17 participants presented with at least one ophthalmological condition;
• 16 participants wore a refractive correction;
• 8 participants had an additional eye condition including amblyopia, visual stress, keratoconus, light sensitivity, Graves Ophthalmopathy and history of an eye trauma;
• 2 participants had undergone eye surgery such as cataract extraction, removal of a corneal ulcer or laser vision correction;
• 4 participants had received eye treatment such as use of eye drops or eye patching in childhood.

This study received ethical approval from The University of Manchester’s Research Ethics Committee (2019-6025-9932) and participants provided informed consent.

### Study Development and Procedure

The research team comprised KP, a Ph.D. student with training in qualitative methods and practicing optometrist by profession; EG, a researcher in the field of sensory perception and motor control in autism; CD, a professor of clinical optometry with a specialist interest in helping those with uncorrectable visual impairment; and CP, a senior lecturer in optometry as well as practicing optometrist with a specialist interest in binocular vision. Across the team existed a wealth of knowledge about the visual system, refraction and ocular health which allowed the research to take a unique approach, as opposed to previous research which has taken a more psychological stance.

The design and procedure of this study were developed in collaboration with the Autism@Manchester Expert by Experience Advisory Group^2^. Thereafter, the research team worked closely with two adult autistic advisors (JP and PB) who ensured an appropriate protocol for the focus groups which would be autism-friendly.

A total of four focus groups were held as this number can reveal up to 90% of all themes (Guest et al., 2017). Each contained four to six participants. Participants were randomly allocated to a focus group depending on their availability to attend. Prior to attendance, participants were sent a “what to expect during the study” document (SM 1) to prepare them for their visit. Upon arrival, they were taken to the focus group room and offered refreshments whilst written consent was taken. Thereafter, they completed a questionnaire which collected basic demographic and diagnosis information as reported above. The focus groups were facilitated by one member of the research team (KP) who followed a predetermined schedule (SM 2). Participants were fully aware that they had access to a quiet room and were able to leave the discussion at any time without having to give a reason. They were also reassured that the data collected during the focus group would be pseudonymized. Another member of the research team (EG or CD) was present to assist with running the sessions which ran for 1-2 h, excluding a short break midway.

In line with recommendations from Durand and Chantler (2014), four key questions were presented to the groups of which three are explored in this paper:

Q1. Does anybody feel they experience any visual problems or unusual visual symptoms?
Q2. Do you feel you can do anything to improve these symptoms?
Q3. How do your visual issues impact your daily routine?

The remaining question (Q4) “what are your experiences of an eye examination?” was unrelated to the topics explored in this paper and will be discussed in a future article. Q1 allowed the researchers to explore the range and magnitude of autistic adults’ visual experiences. A key aim of this study was to characterize these experiences in detail by understanding what steps autistic adults take to tackle these (Q2) and what affect they have on an autistic adults’ life (Q3).

### Data Analysis

The focus groups were audio recorded and then transcribed, with participants pseudonymized, by an external university approved service for intelligent verbatim transcription. Transcripts were thematically analyzed to allow the broad range of data to be brought into meaningful themes. Compared to other qualitative analysis methods, thematic analysis allows data sets to be richly described as a whole and goes further than just summarizing data (Braun and Clarke, 2006; Maguire and Delahunt, 2017). The analysis aimed to be exploratory and the research student (KP) took an inductive, semantic and realist approach from the point of a non-autistic optometrist.

The Braun and Clarke six-step technique (Braun and Clarke, 2006) was followed as this framework is flexible and can be easily applied to a variety of research questions. Firstly, the accuracy of each transcript was checked against the original recordings. The research student then familiarized himself with the data by re-reading through the transcripts whilst making any initial notes of key ideas. The second phase involved re-reading and line-by-line coding of the transcripts to identify features (words, sentences or paragraphs) of the data related to the scope of the study. This was done by hand and codes were written on sticky notes.

In the third phase, codes were grouped to form initial themes. For this, as per the recommendations of Braun and Clarke (2006), a physical thematic map was created by arranging the sticky notes according to similarity in content or ideas. This allowed the research student to visualize the formation of higher-level themes. These three stages were followed for each transcript and moderate alterations were made to the thematic map as more transcripts were analyzed.

Data saturation was reached with no new themes developing from the fourth focus group. The fourth phase reviewed the allocated themes against the dataset as a whole. It was important that the themes captured all relevant aspects of the data. The themes and codes were discussed amongst the research team (KP, CD, CP, and EG) to improve the rigor of the analysis and ensure a valid interpretation of the data. The team agreed that the codes summarized the relevant aspects of the data well, however, some themes could be grouped together as they were (a) very small and (b) closely related. The thematic map was reorganized as per these modifications (see Table 1).

**TABLE 1:** The six allocated themes and their definitions as well as respective subthemes, grouped according to the question from which they arose.

**Question 1: Does anybody feel they experience any visual problems or unusual visual symptoms?**		
***Themes***	***Theme definition***	***Sub-themes***
Altered visual	Visual symptoms or unusual	Visual hypersensitivities
experiences	occurrences experienced by	Eye movements
	participants	Visual experiences vary from person to person
Autistic individuals’ vision-related knowledge	The level of understanding participants had surrounding their vision and ocular health, and the impact of this	Degree of awareness Impact of awareness

**Question 2: Do you feel you can do anything to improve these symptoms?**		
Coping strategies	Methods adopted by	Avoiding visual clutter
	participants to tackle their	Optical correction choices
	visual experiences	Colored overlays/lenses
		Lighting alterations
		Just cope with it
		A multisensory experience

**Question 3: How do your visual issues impact your daily routine?**		
Impact on personal	The multi-dimensional impact of	Physical wellbeing
wellbeing	visual experiences on	Mental wellbeing
	participant wellbeing	Emotional wellbeing
Impact on daily life	The impact of visual	Home life
	experiences on participants’	Work life
	daily lives	Public places
		Travel
		Social life
A poor public understanding of sensory issues in autism	The perception of a poor awareness in the general population surrounding autism and the participants’ reaction to this	

Themes were appropriately named and given a short definition in the fifth phase. The research team had to ensure that a theme’s name gave an immediate reflection of what was covered therein and highlighted its relevance to the scope of the study. Additionally, a detailed analysis of each theme, most either complex or large, lead to the allocation of multiple subthemes. The final phase involved bringing together the themes and supporting data in a report. For this, appropriate quotes were chosen from the data set which justified the research findings (see section “Results”), and the overall outcomes needed to be discussed in the context of the study aims and existing literature (see section “Discussion”).

## Results

A final six themes were allocated to the data and are listed in Table 1 under the question from which they arose; themes, theme definitions and corresponding subthemes are presented.

The remainder of this section describes these themes in further detail. The participants are referred to by a number (P1-18).

### Theme 1: Altered Visual Experiences

#### Visual Hypersensitivities

Participants described a variety of issues, relating to visual hypersensitivity, which refers to an increased sensory sensitivity rather than threshold detection sensitivity. Hypersensitivity to lighting had multiple aspects; bright, flickering, fluorescent, “strip,” and “spot” lighting caused discomfort. Participants, with and without a diagnosis of visual stress, described difficulties reading and viewing certain patterns. P4, who suffered with visual stress, said, *“*…*it’s like, the letters*… *flicker around the edges sometimes. As if the letters are bleaching into the gray bits*…*”* which describes typical visual stress characteristics. Our participants also portrayed visual stress to be caused by day-to-day striped visual images such as “grills on buildings” or “radiators.” P9 explained an adverse response to patterns, again characteristic of visual stress, although they did not have a formal diagnosis:

*“*…*I definitely sometimes have what feels like a physiological response to the patterns*…*It’s like you’ve been punched in the stomach. It’s a real strong emotion that comes over you like when there’s loud noise.”*

Hypersensitivity to particular colors could cause an adverse response, *“*…*I get like a physical reaction to them as though they’ve hit you”* (P9), and was suggested to largely influence our participants’ likes and dislikes, where they visited and what they selected. *“I mean I really don’t like the color yellow. I mean the railing down there is toddler screaming levels of irritation. And I’m not fond of bright reds”* (P8); “…*all of my upholstery I’ve chosen, it’s beige*… *But for me, I mean it’s not that nice to look at*… *But if I had any bright colors then I would just avoid that room”* (P11). Participants indicated that the impact of color cannot be predicted because the contrast with the surroundings, the pattern formed with other colors and the combination of colors are all influential factors.

Hypersensitivity to visual motion occurred mostly in crowded, busy environments and was implied to be due to a combination of visual clutter and movement. P14 explained:

*“It’s the movement of other people, because to me it’s*…*if I’m in my living room, my children are running back and forth across, I find that very stressful. They need to be on one side, not going across my field of view all the time. I get stressed and angry.”*

The main impact of visual hypersensitivity was distraction. P1 said:

*“*…*the general theme of being distracted by visual things is an issue for me. Especially because I wear glasses and they always get dirt on; I notice the dirt a lot more than other people.”*

Participants suggested they were more aware of their full field of view and had difficulty paying attention to a particular part of it. A conscious effort has to be made to overcome distraction, *“I’m not at the moment looking the other way, but if I was tired, I’d be much more distracted. So, it’s almost like playing a filter to try and filter it all consciously”* (P5).

The inability to overcome distraction caused negative emotional responses such as anger and frustration, *“*…*it drives me mad*…*”* (P5). However, not all distractions had a negative impact. Some participants said they occasionally dedicated their attention to one visual stimulus which fully occupied their sensory system to prevent distraction from other stimuli, *“*…*so I can ignore the sounds and the visual stimulus of people talking, I focus as firmly as I can on what I’m reading”* (P8).

#### Eye Movements

Four participants expressed difficulties with controlling eye movements. Others had been made aware of problems with eye tracking through research studies requiring eye calibration which suggests that these issues are not always apparent to autistic individuals. P1 reported, *“My eye tracking is a bit weird. I think I don’t look at*…*the thing I’m intending to look at sometimes or following it correctly”* and confirmed this to be the case *“*…*all the time to some extent.”* P13, who did not declare any binocular vision problems, stated:

*“*…*I hadn’t realized how much I’d struggled [with reading] for years, because it’s sort of double, but it’s very subtly double. And sometimes it just goes like that [hand gesture indicating diplopia].”*

#### Visual Experiences Vary From Person to Person

Participants’ visual experiences varied from person to person depending on the severity of their altered visual sensory sensitivity. For example, P7 said:

*“*…*I have more issues with sound. Not excessively, as sometimes you hear about, but certainly more so with sound than with vision. Which is why, like I say, I couldn’t really relate to what was being discussed*…*”*

Many participants felt they had good vision and could *“see clearly”* (P14), however, some felt their eyesight was genuinely poor. P15 said, *“*…*I never feel I can see well enough, ever.”* Participants were quick to suggest that they could differentiate perceptual symptoms from sight issues, *“I don’t think my eyes see differently from other people, I think I process it differently”* (P1).

### Theme 2: Autistic Individuals’ Vision-Related Knowledge

#### Degree of Awareness

The degree of awareness about vision and eye health varied across the participants. They expressed little flexibility with their definition of good vision, thinking 20/20 acuity and a low spectacle prescription defined this. The presence of non-pathological floaters, the time taken for light adaptation, and foveal bleaching as a result of viewing a bright object all seemed to be perceived as poor eye performance by participants although these are normal physiological phenomena, *“So the floaters are the big thing*… *At work, the dark floaters at work, that’s not good vision”* (P6).

#### Impact of Awareness

The degree of understanding that participants had about these aspects in turn impacted their emotional response to them. In cases where there seemed to be a lack of understanding, participants expressed negative emotions such as fear, anxiety and feeling abnormal, “*I just feel like I’m made up of bad code*” (P6). Well informed participants appeared to be less concerned about the same experiences.

### Theme 3: Coping Strategies

#### Avoiding Visual Clutter

Participants indicated that visually busy environments overwhelmed their sensory system, so tended to avoid them. P3 said, *“*…*the city center, being surrounded by buildings and people like it’s all too much visual information.”* Where avoidance was not possible participants minimized the time they had to experience visual clutter, *“I tend to use the same shops. So I know exactly where things were last time, barring the usual rearrangements. So I try and get it done quickly. So I can get out of there”* (P8).

#### Optical Correction Choices

Interestingly, participants discussed their choice of spectacles. P1 said, *“different glasses would help me, like if they were rimless or I wasn’t distracted by a frame*…*”* Although different participants interpreted the effect of the frame size in different ways, the general conclusion was to avoid seeing the rim, *“you’re physically aware of them, this is slightly annoying”* (P7). Participants requested more reassurance when being dispensed new spectacles and identified the need for a relatively longer adaptation period to these.

Although sunglasses and photochromic lenses are helpful as they reduce light levels, they were suggested to be only a partial solution. P8 stated, *“*…*it’s a little disconcerting having that artificial darkening when I’m used to things seeming bright*,*”* which leads to the issue of feeling *“detached”* (P10). It appears that this population may struggle with optimal light levels without visual sensory issues being compromising.

#### Colored Overlays/Lenses

The use of colored overlays and lenses was beneficial for some participants who experience visual stress symptoms. P13 stated, *“*…*my reading speed doubled*…*I could actually see properly.”* P2 said, *“*…*I never saw the social world, I never saw people, never saw expression*…*”* prior to using colored lenses. However, they were not equally beneficial for all. P14 said her tinted lenses were *“*…*not all that helpful with the visual stress. Just good with headlights and blueness.”*

#### Lighting Alterations

Light alterations increased participants’ ability to cope in artificially lit environments. Reducing light levels can improve visual ability, *“*…*I can see very well in low light”* (P8). Whereas natural color temperatures, such as ‘daylight’ ease visual symptoms and are *“preferred”* (P1), warmer color temperatures are *“very warm, very comforting”* (P4). Some participants also suggested that blue blocking lenses *“relax”* (P10) them and make spectacles feel better.

#### Just Cope With It

The final approach was to *“cope, cope as best as you can”* (P9). Participants described that as a hypersensitivity is increasingly provoked it induces a greater negative emotional response, leading to growing distress. Apart from being stressed and anxious, they are likely to inconvenience themselves by trying to prepare for every situation, *“I just carry loads of different types of glasses with me*… *So it just means I’m covered for all eventualities*…*”* (P13).

#### A Multisensory Experience

It was challenging for some participants to think about vision-related coping strategies because visual sensory symptoms usually occurred as part of a multisensory experience for them:

*“*…*it’s difficult to pull out that that is due to any particular reason really. I mean the talking to people I struggle with if there’s the stripy shirt distraction issue, because it’s something that’s grabbing your attention away from what they’re saying. At the same point as there’s five different people behind them whose conversations you’re listening to at the same time because you can’t screen them out. So it’s difficult, isn’t it, to say what is due to which issue.”* (P9).

### Theme 4: Impact on Personal Wellbeing

Altered visual sensory reactivity had multiple impacts on our participants’ wellbeing.

#### Physical Wellbeing

Physically, visual experiences are *“a gradually fatiguing thing”* (P10), which impacted the participants’ functionality. P14 said, *“flickering lights, like the sun behind trees, makes me sleepy.”* Additionally, many participants expressed sleeping difficulties, especially during summer months due to longer daylight hours.

#### Mental Wellbeing

Participants lacked a feeling of self-worth due to low self-confidence as expressed by P6, *“I feel like my genetics are just really bad codes, just full of defects and errors. And that’s [referring to their vision] just another error to catalog.”* P4 suggested that this lack of confidence could be a result of not knowing *“what other people see”* which constantly makes them doubt if they are seeing the world in the same way as non-autistic individuals.

#### Emotional Wellbeing

Emotional wellbeing varied amongst our participants. P1 said, *“it [visual hypersensitivity] just makes me generally stressed all the time and less able to deal with other things*.*”* P6 was angry about his vision, *“I’m not happy with my eyesight at all. No, I’m not,”* whereas P12 found his vision simply *“overwhelming*.*”* Conversely, participants who saw the advantages of their visual experiences portrayed themselves as relatively more positive in the way they spoke, their responses and their body language, as explored in the next theme.

### Theme 5: Impact on Daily Life

#### Home Life

Visual experiences were a hindrance in home life for some of our participants, especially for tasks requiring concentration such as *“cooking and sewing”* (P1); these can be difficult to complete with ease and enjoyment. Some participants, however, saw their visual hypersensitivities as an advantage especially for hobbies. P4 stated:

*“*…*seeing details is a double-edged sword. On the one hand you could get overwhelmed with all the detail. But at the same time, it also means*… *When I’m painting, I can see far more detail than other people can see. I can spot things that other people miss.”*

#### Work Life

Regarding employment, participants expressed positive views. In particular, sensitivity to fine detail was an advantage at work:

*“*…*I just use my ability to pay attention to visual detail more, more in my professional work which involves a lot of image analysis and data processing to produce those images*…*”* (P2).

#### Public Places

Having to avoid certain environments due to visual hypersensitivities meant participants were more likely to stay at home. They expressed issues with cinemas, large shops, hospitals and lecture theaters specifically. However, issues with public places cannot solely be blamed on visual experiences. Participants recognized that these difficulties are more due to multisensory problems and anxiety which collectively overwhelms them:

*“it’s a multi-level thing. I mean hard, easy to clean floors, which means every person stepping around is bang, bang, bang. You’ve got people moving around randomly. You’ve got the bright lights. and you’ve got all the people talking simultaneously.”* (P8).

#### Travel

Participants said they can be distracted and overwhelmed by visual clutter, headlights and objects that catch their attention when driving. Some had given up driving due to their visual experiences, whereas others had not pursued driving due to a fear of these:

*“I mean headlights are a problem when driving in the dark. It’s like the headlights seem to bleed and wash out some of the rest of the visual experience, and you need that data when you’re driving in the dark.”* (P10).

Participants found it difficult to use public transport due to the artificial lighting at night or sunshine on bright days. The majority of participants in our study suggested that the issues with public transport were multidimensional and again could not be accounted for just with vision:

*“*…*dealing with the driver, walking past all the people to find a seat and finding a seat with the person next to me*… *And then just the overall noise and the rattling of the engine and the windows”* (P10).

#### Social Life

Many participants felt that sensory experiences contributed to difficulties in their social life. When asked about inclination to attend social events, P4 said, *“I think less inclined purely because I don’t want the overload of all the sensory input*.*”* As described by P1, the stress induced by visual experiences reduces their ability to deal with other situations such as *“interacting with people*.*”* Due to these experiences, P3 felt limited in her social life:

*“I drive because I can’t do public transport. And so, if we think about the impact socially and stuff then a lot. So, I can’t go out drinking because I have to drive home and stuff like that as well.”*

However, our participants’ difficulties and limitations in social situations could be misinterpreted as being *“antisocial”* (P4).

### Theme 6: A Poor Public Understanding of Sensory Issues in Autism

Participants described a lack of awareness in the general population regarding the sensory difficulties autistic adults face. P5 felt the ignorance of some non-autistic individuals toward autistic people is *“absolutely disgusting.”*

Educating the general public about the sensory issues in autism is important to heighten understanding, *“*… *now my wife understands why I have to leave things early. Before she just thought I was being antisocial. Now she knows she’s more understanding about it*” (P4).

There was also a fear amongst our participants that their difficulties may not be understood by employers or public services. For example, P10 said:

*“*…*I’m really dreading I think to have some kind of sensory conversation with an employer. Because there’s some environments I go into now, like the hospital, and I don’t think I could physically tolerate that. So yeah, I think this has big implications*…*”*

## Discussion

The current study is the first to provide an in-depth qualitative investigation of autistic visual experiences, together with their impacts on daily life and coping strategies. A total of 18 autistic adults, without learning disabilities, attended a focus group meeting at The University of Manchester. The opinions of these participants were elicited to gain a holistic understanding of the visual experiences of the autistic adult population. It builds on previous work which has briefly documented visual issues in the context of a broader study on altered sensory reactivity (Jones et al., 2003; Robertson, 2012; Robledo et al., 2012; Smith and Sharp, 2013; Robertson and Simmons, 2015), and highlights significant concerns amongst autistic adults regarding their vision, visual sensory experiences and the impacts these have.

### Characteristics of Visual Issues

As noted by previous literature, our participants highlighted increased sensitivity to different aspects of lighting (Bogdashina, 2003; Leekam et al., 2007; Robledo et al., 2012) and fine detail (Simmons et al., 2009; Kabatas et al., 2015). Participants discussed strong likes and dislikes for particular colors, agreeing with the findings of a case report by Ludlow and Wilkins (2009). This also appears to be analogous to the outcomes of Grandgeorge and Masataka (2016) who investigated color preference in autistic boys aged between 4 and 17 years, finding they were significantly less likely to prefer yellow, compared to age-matched controls, but more likely to prefer green and browns. Such color preferences were suggested to be a result of autistic visual hypersensitivities, also indicated by our participants.

Additionally, participants reported key symptoms of visual stress when viewing repetitive patterns, including flickering, fading and a strong discomfort (Evans and Stevenson, 2008). Although a few participants described the impact of this phenomena particularly with reading, it was largely discussed as a global experience impacting several aspects of daily life, and dependent on the combination of colors which produced an uncomfortable contrast. Robertson and Simmons (2015) also found their focus group members to describe visual stress symptoms in the context of more global aspects such as the pattern formed by the layout of products on shelves in a shop. In view of this and other studies reporting first-hand accounts of pattern sensitivity in autistic adults, future work should investigate whether the visual experiences of autistic people are at all related to Meares-Irlen syndrome.

Our participants suggested issues with eye tracking, visual location and control of binocular vision (reporting diplopia), but it is unclear how these reports would relate to formal laboratory measurements reported in the literature. A review and meta-analysis by Johnson et al. (2016) reported that autistic individuals can have altered eye movements, specifically poor eye tracking, impaired saccade inhibition and saccade dysmetria, but do not have difficulty initiating saccades or engaging/disengaging from targets. Additionally, studies have found autistic people to make faster eye movements during predictive saccade tasks (D’Cruz et al., 2009; Kovarski et al., 2019).

Autistic individuals are more likely to develop ophthalmological conditions (Little, 2018). These include refractive error, binocular vision and ocular muscle balance anomalies, and altered retinal structure. It is not known whether any of these deficits may contribute to autistic sensory symptoms although this would be a valuable relationship to investigate.

It is important to note that visual sensory experiences varied amongst our participants, depending on how sensitive they were to their vision. For example, those more sensitive to sound did not necessarily fully relate to the accounts of participants reporting severe visual symptoms. This variability is evident in existing research on autistic altered sensory reactivity and highlights the importance of not merely generalizing findings across the autistic adult population.

### Visual Sensory Experiences and Attention

Autistic individuals have displayed and described impairments with attention (Liss et al., 2006); Patten and Watson (2011) discussed alterations in three broad features of attention in autistic children: orientating, sustaining and shifting. Attention is closely linked with distraction and previous studies have demonstrated that autistic individuals have difficulty ignoring irrelevant distracting sensory information (Christ et al., 2011; Adams and Jarrold, 2012; Smith and Sharp, 2013). Reasons for this could be greater perceptual capacity (Remington et al., 2009; Bayliss and Kritikos, 2011; Tillmann and Swettenham, 2017) or enhanced sensory sensitivity (Liss et al., 2006), both of which have been found to be positively correlated to each other in a recent study by Brinkert and Remington (2020). Our participants indicated that they had to make a conscious effort to attend to their central field of view and ignore their peripheral vision, in agreement with Mottron et al. (2007) who investigated lateral glances in autism.

Difficulties with distraction led to issues with driving for our participants. The visual sensory experiences encountered during driving can be overwhelming and autistic adults can struggle to pay attention where it is required. The literature suggests this population display relatively more problematic driving behaviors (Daly et al., 2014), and are less likely to attend to all relevant parts of their visual field during driving. However, visual issues can be one of many aspects which impact driving ability (Reimer et al., 2013). Autistic individuals are more prone to becoming anxious (Reimer et al., 2013), and have shown difficulties with motor coordination, staying in lane, control of speed, and adapting to unexpected situations during driving (Classen et al., 2013).

### Multiple Impacts of Visual Sensory Experiences

Our study suggests that visual experiences can contribute to difficulties maintaining emotional, mental and also physical wellbeing. As well as causing pain and negative physiological responses, fatigue caused by altered visual sensory reactivity appeared to directly impact on the functioning of our participants. Emotionally and mentally, our participants largely expressed low mood and negative feelings, such as fear and stress, due to their visual experiences. They saw themselves as excluded because of the sensory problems they faced, which included vision.

Visual experiences could contribute to poorer daily living skills that are present in autistic individuals (Smith et al., 2012; Bal et al., 2015). Chores in the household such as cooking, and visiting public places including shops and hospitals, were all made more difficult as a result of visual experiences for our participants. They suggested being put off tasks which demand a lot of visual attention; visual experiences limit them to a few tasks which they can complete and enjoy.

However, sensory experiences can have positive aspects too. They can be enjoyable specifically when they or the associated anxieties are under control (Jones et al., 2003). Our study found that hypersensitivity to fine detail can prove an advantage to autistic people as they can detect details which non-autistic individuals may overlook. Although this was the case, participants did not mention this to be related to any positive effect on their mental or emotional state as also noted by Robertson and Simmons (2015). Robledo et al. (2012) also observed that some participants enjoyed visual stimulations such as bright lights and particular color combinations. Seeking the positives in sensory experiences was identified as a coping strategy by Jones et al. (2003).

### Coping With Visual Sensory Experiences

Little research has been carried out to date to investigate autistic individuals’ coping strategies for their sensory issues (Jones et al., 2003; Smith and Sharp, 2013; Robertson and Simmons, 2015). Our results agree with Smith and Sharp (2013) who found that moderating factors, such as reduced sensory inputs, reduced sensory intensity, predictable environments and the autistic person being calm, can lower the impact of otherwise overwhelming sensory experiences. Our participants suggested that autistic adults can feel overwhelmed by a large variety of visual information. They attempted to prevent sensory overload by means such as avoiding visually cluttered public places at peak times and shopping at the same stores as they would know where items are kept.

The effort made by participants to prevent sensory overload by avoiding social interaction could be misunderstood as awkwardness or being uncooperative. Participants were disappointed and anxious about the poor public understanding of autism and associated sensory issues. They agreed that this has to be improved, which agrees with recent recommendations in The Autism Dividend report (Lemmi et al., 2017). Our study has attempted to describe the visual experiences of autistic adults without learning disabilities so that professionals, service providers and members of the public can develop an understanding of this and be more accommodating.

Altering lighting, in terms of brightness and color temperature, was also beneficial for our participants and felt to improve visual performance. This could be related to visual stress with these light adaptations having a similar effect to the use of colored overlays or lenses. Participants also commented on the relaxing nature of blue blocking lenses, agreeing with a randomized trial in non-autistic individuals (Kimberly and James, 2009) which concluded that these can significantly improve mood.

The benefits of colored overlays or lenses in autism have been speculated upon. Some studies have shown improved reading speed (Ludlow et al., 2008; Ludlow and Wilkins, 2009), better control of behavior, coordination and personal space (Ludlow and Wilkins, 2009), and improved ability to characterize the intensity of facial expressions (Whitaker et al., 2016). Some of these social aspects were confirmed by the personal accounts of our participants. Though colored lenses reduced visual stress for some of our participants, for others they did not, and no rigorous controlled trials have yet been conducted in this area. It is therefore crucial for optometrists and autistic individuals to know that while there may be possible benefits of prescribing colored lenses, they may not work as expected in all instances and further research is needed.

The final approach to “just cope” resulted in participants experiencing a variety of negative emotions. As per Carver et al. (1989), there are two distinct forms of coping: problem focused and emotion focused coping. In terms of our findings, although participants’ coping strategies could be grouped as one or the other, we do not know how they reached these stages.

Some of these coping strategies could be underpinned by “compensatory mechanisms,” which involves alternative cognition to bypass cognitive difficulties. As a result, autistic people display fewer behavioral symptoms despite continued underlying cognitive and neural deficits. These mechanisms can be applied to compensate for particular cognitive atypicalities, as opposed to “camouflaging” which aims to mask all autistic traits (Livingston and Happé, 2017; Livingston et al., 2019). It is therefore not surprising that Robertson and Simmons (2015) suggest specific coping strategies developed by autistic adults could help explain some of the unusual behaviors adopted by this population. The overall message from our results is that visual experiences result in a variety of issues for autistic adults which result in strong positive and negative emotional impacts. A range of coping strategies are employed to deal with these.

### Participant Interpretations of Visual Sensory Experiences

Our participants thought that many of their visual experiences were a result of higher-level processing issues and not necessarily due to uncorrected refractive error or poor binocular vision. However, the degree of vision-related knowledge varied amongst our participants and appeared to influence anxiety about their visual issues. For example, some were worried, feeling normal ocular phenomena were a sign of poor eye performance while the opposite was true for those who had a good understanding of phenomena such as light adaptation and floaters. It is important to note that health-related anxiety is likely to vary similarly in the general population too so we cannot conclude that this is an issue confined to autistic adults. Nevertheless, to reduce this anxiety in autistic individuals, there is a need to increase their understanding around vision and eye-health.

Moreover, participants indicated that their visual experiences usually occur as part of a larger multisensory experience which may be a reason as to why they generally found it difficult to specify the contribution of vision to their sensory experiences. Issues with public transport are a good example of this; hypersensitivity to light is one aspect, but this is part of a multisensory issue alongside anxiety. Indeed, processing multisensory stimuli is altered in autism (Robertson and Baron-Cohen, 2017). It may be the case that altered sensory processing in one modality has an impact on other modalities. This could amplify or dampen the sensory symptoms.

### Limitations and Considerations

To our knowledge, this is the first in-depth qualitative study which set out to explore the subjective visual issues experienced by autistic adults, the impacts these have on their daily lives and what they do to minimize these. However, our results can only be considered for autistic adults without learning disabilities. Those with coexisting learning disabilities or other neurodevelopmental disorders may also experience visual sensory symptoms: an observational study for individuals who cannot express their symptoms verbally may identify corresponding behavioral signs. Additionally, participants were limited to those who could communicate in a focus group setting. A further study offering interviews or online focus groups for individuals who cannot take part in a physical verbal group discussion may have yielded further insights. Individuals may have been more likely to participate in this study if they were aware of having visual problems which could have resulted in reports of more negative or extreme experiences. However, as our aim was to describe visual experiences rather than quantify them this has less of an impact on our results.

Sample size determination is difficult in qualitative research and there are alternative approaches suggested for this. A recent article by Braun and Clarke (2019) discusses data saturation in the context of thematic analysis and suggests that it is difficult to justify sample size with data saturation for studies which aim to be exploratory, inductive and that do not ask exactly the same questions during every focus group. In our study, recommendations regarding number of focus groups by Guest et al. (2017) and data saturation, during the planning and data analysis phases respectively, were used to confirm a suitable sample size. However, in line with suggestions by Braun and Clarke (2019), our focus groups were on a very select topic and all of our participants were autistic and had experience of an eye examination, therefore each was likely to have more “information power” (Malterud et al., 2016), meaning our modest sample size was acceptable.

Many of our participants also had coexisting conditions such as dyspraxia, ADHD and anxiety disorder which may have influenced our results. There is evidence that individuals with dyspraxia have defective global spatial processing (O’Brien et al., 2002). Mogg et al. (2000) concluded that individuals with generalized anxiety disorder display altered eye movements to threatening facial expressions. In a national United States investigation, there was a greater prevalence of ADHD among children with visual problems which could not be corrected with spectacles or contact lenses (DeCarlo et al., 2016). Although it would be interesting for future work to identify if there are autism specific visual experiences, including individuals with these co-occurring conditions, due to their high prevalence in autism, is relevant for providing a realistic description (Gillberg and Billstedt, 2000).

## Conclusion

This study provides a first-hand insight into the range of visual issues and their impacts within the autistic adult community which cannot be expressed through objective or quantitative studies. The findings have confirmed that autistic adults are often dissatisfied with their vision and experience a range of visual sensory symptoms which vary from person to person. These symptoms can occur alone or as part of a larger multisensory response, nevertheless, vision contributes to sensory issues and emotional responses. It is noteworthy that although some of the visual experiences expressed by our participants can be expected to occur in non-autistic people, it was the magnitude, frequency and impact of these experiences which was unique and suggested to be greater. Although a large part of the visual experiences suggest issues with higher level processing, there is indication that some symptoms associated with control of binocular vision and visual stress could benefit from an optometric assessment. Finally, autistic adults employ a variety of strategies to overcome their visual symptoms, but the last resort is to endure these.

## Data Availability Statement

The original contributions presented in the study are included in the article/Supplementary Material, further inquiries can be directed to the corresponding author/s.

## Ethics Statement

The studies involving human participants were reviewed and approved by The University of Manchester’s Research Ethics Committee (UREC). The patients/participants provided their written informed consent to participate in this study. Written informed consent was obtained from the individual(s) for the publication of any potentially identifiable images or data included in this article.

## Author Contributions

KP, EG, CD, and CP led the study, designed the study, recruited the participants, conducted the data analysis, and wrote the manuscript all with the support of his supervisors. EG, CD, and CP were involved in designing the study and finalized the data analysis themes. EG and CD supported the focus group sessions which were facilitated by KP. JP and PB were advisors for the duration of this study and gave suggestions to ensure the study was accessible to autistic adults. All authors were involved in editing the manuscript and approved the submitted version.

## Conflict of Interest

The authors declare that the research was conducted in the absence of any commercial or financial relationships that could be construed as a potential conflict of interest.

## Footnotes

1 https://www.read123.co.uk/en/the-use-of-color-therapy-and-colored-lenses-in-autism/

2 www.autism.manchester.ac.uk/connect/expert-by-experience
