# Supplementary material for: Visual Sensory Experiences From the Viewpoint of Autistic Adults
Source: Front Psychol. 2021 Jun 8;12:633037. doi: 10.3389/fpsyg.2021.633037 (PMC8217662; doi:10.3389/fpsyg.2021.633037)
Supplement: Supplementary file 1 [file Table_1.DOCX]

Supplementary material

**SM 2: The focus group schedule**

Introduction and purpose of focus group

Hello. My name is Ketan Parmar. I’d like to start off by thanking each of you for taking time to participate today. We’ll be here for about an hour.

The reason we’re here today is to gather your opinions and thoughts on the visual symptoms autistic individuals may experience, how these can impact your daily life and any experience you have had of an eye examination.

I’m going to lead our discussion today. I will be asking you questions and then encouraging and moderating our discussion.

I also would like you to know this focus group will be recorded as was mentioned in the participant information sheet. The identities of all participants will remain confidential. The recording allows us to revisit our discussion for analysis and writing up the results into a paper. I will be able to engage more with you as opposed to writing constantly whilst you speak.

Ground rules

To allow our conversations to flow more freely, I’d like to go over some ground rules.

1. Only one person speaks at a time. This is doubly important as our goal is to make a written transcript of our conversation today. It is difficult to capture everyone’s experience and perspective on our audio recording if there are multiple voices at once.
2. Please avoid side conversations.
3. Everyone doesn’t have to answer every single question, but I’d like to hear from each of you today as the discussion progresses.
4. This is a confidential discussion in that I will not report your names or who said what to any outside of this room. Names of participants will not even be included in the final report about this meeting. It also means, except for the report that will be written, what is said in this room stays in this room.
5. We stress confidentiality because we want an open discussion. We want all of you to feel free to comment on each other’s remarks without fear your comments will be repeated later and possibly taken out of context.
6. There are no “wrong answers,” just different opinions. Say what is true for you, even if you’re the only one who feels that way. Don’t let the group sway you. But if you do change your mind, let me know.
7. Let me know if you need a break.
8. Are there any questions?

Introduction of participants

Before we start the discussion and recording can we go around the room and introduce ourselves with our names and where we have come from today.

Focus group discussion

*Begin recording*

1. Does anybody feel they experience any visual problems or unusual visual symptoms?
   1. Can you give me an example?
   2. Do you experience these in a particular environment or time of day?
   3. Do you feel you see as well as your colleagues/ friends/ relatives?
   4. Is your vision clear? Do you ever feel it could be better?
   5. Do you notice any visual problems when doing particular tasks? Reading? Stitching? Computer work?
   6. Is there anything that you can’t visually tolerate?
   7. Is there anything that makes your vision uncomfortable?
2. Do you feel you can do anything to improve these symptoms?
   1. Taking breaks from VDUs?
   2. Avoiding particular environments? Making changes to your home/ work environment?
   3. Avoiding particular colours?
   4. Avoiding particular patterns?
   5. Wearing glasses? Sunglasses?
   6. Eye treatment, e.g. drops?
3. How do your visual issues impact your daily routine?
   1. Work?
   2. Social life?
   3. Commuting?
   4. Communicating with others?
   5. Using equipment and completing tasks?
   6. Fear, stress or anxiety inducing?
   7. Do you feel your vision is contributing to any other issues you may face?
   8. Danger inducing?
4. What are your experiences of eye examinations? *(positives and negatives)*
   1. The process of booking your appointment.
   2. The practice environment.
   3. The testing room.
   4. The tests.
   5. How the practitioner spoke to you-did you understand it all?
   6. Any suggestions that could have resulted in a more efficient and successful eye examination?
   7. Some tests require the practitioner to get very close; how can we better relax you for these?
   8. If you haven’t been for an eye examination, is there a reason? Have you been put off?

Prompts and probes:

- Has anyone else had this experience?
- What else do you dislike?
- Thank you for your contribution; does anyone else feel as strongly about this?
- In what way?
- I don’t understand?
- You seem confused; what don’t you understand?
- What do others think?
- Can you tell us more?

Closing

Thank you all for your time in attending today and discussing your opinions on the questions I presented. Your discussion has been very useful for the progression of this project.
