# Supplementary material for: Visual Sensory Experiences From the Viewpoint of Autistic Adults
Source: Front Psychol. 2021 Jun 8;12:633037. doi: 10.3389/fpsyg.2021.633037 (PMC8217662; doi:10.3389/fpsyg.2021.633037)
Supplement: Supplementary file 2 [file Table_2.DOCX]

Supplementary material

**SM 1: “what to expect during the study” document**


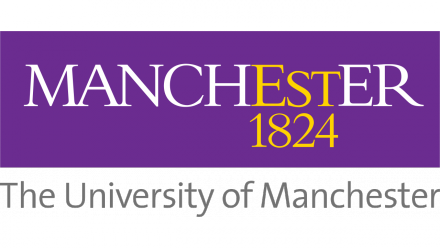


**Investigating optometric and orthoptic conditions in autistic adults: Part 1**

**Focus groups**

What to expect when you attend

Thank you for agreeing to take part in this study. This document gives you some detail on what to expect when you attend for the focus group session and how the day will run.

The focus group will take place in the Carys Bannister building at the University of Manchester. This is located on Dover Street off Oxford road; please see the map image below.

[Map showing location of the Carys Bannister building]

The main entrance to the Carys Bannister building is through the revolving doors on Rumford street (this is just off Dover street; see map). On arrival you will be greeted by Ketan who will help you sign in and escort you to the meeting room where the focus group will take place. There will be some refreshments provided too, prior to the meeting and available during the meeting if you require. There will also be a separate “quiet room” that you can sit in at any point if you choose to – please let Ketan know either before or on the day if you would like to use this.

The focus group room

[An image of the focus group room with a large round table and chairs]

If you will be driving into the University for the focus group and require parking please let Ketan know 2-3 days prior to your scheduled focus group meeting so that he can arrange this for you.

You will have the opportunity to re-read the “participant information sheet”, if you wish, and ask any questions. We will thereafter ask you to complete a consent form.

Once you have given your consent to take part in this study you will be asked to fill in a “participant information form”. This asks for some basic details and also more specific information regarding your autism. Please ensure you bring your diagnosis letter with you to help you complete this.

When all participants have had the opportunity to ask any questions, provided consent and completed the “participant information form”, the focus group will begin. You can choose where you sit.

The quiet rooms

[Two images of rooms, separate from the focus group room, which would serve as “quiet rooms”]

There will be two members of the research team in the room too. Ketan, the student researcher will begin and facilitate the focus group. Please note that the meeting will be audio recorded as this is important for us to be able to analyse the research. Ketan will say when the recording will begin.

The focus group will be an informal discussion about your vision. Ketan will ask some key questions to begin the discussion and to ensure we remain in line with the aims of the meeting.

If at any point in the focus group you feel uncomfortable or need a break, you can ask to pause the recording or be taken to the quiet room. When you are ready to re-join you will be brought back to the focus group room.

On completion of the focus group you will be given a small reimbursement for your time; please bring along your travel expense ticket/ receipt so that we can reimburse this too. You will be at the University for approximately 1½ hours.

[Image of KRP]

We hope this document has given you a good understanding of what the focus group session will be like. If you have any further questions you can contact Ketan by email (*email address*) or ask upon arrival at the focus group.

Ketan Parmar
